# Somatosensory Representations Link the Perception of Emotional Expressions and Sensory Experience^1^,^2^,^3^

**DOI:** 10.1523/ENEURO.0090-15.2016

**Published:** 2016-04-29

**Authors:** Philip A. Kragel, Kevin S. LaBar

**Affiliations:** Department of Psychology & Neuroscience and Center for Cognitive Neuroscience, Duke University, Durham, North Carolina 27708

**Keywords:** embodied cognition, emotion, functional magnetic resonance imaging, perception, somatosensation

## Abstract

Studies of human emotion perception have linked a distributed set of brain regions to the recognition of emotion in facial, vocal, and body expressions. In particular, lesions to somatosensory cortex in the right hemisphere have been shown to impair recognition of facial and vocal expressions of emotion. Although these findings suggest that somatosensory cortex represents body states associated with distinct emotions, such as a furrowed brow or gaping jaw, functional evidence directly linking somatosensory activity and subjective experience during emotion perception is critically lacking. Using functional magnetic resonance imaging and multivariate decoding techniques, we show that perceiving vocal and facial expressions of emotion yields hemodynamic activity in right somatosensory cortex that discriminates among emotion categories, exhibits somatotopic organization, and tracks self-reported sensory experience. The findings both support embodied accounts of emotion and provide mechanistic insight into how emotional expressions are capable of biasing subjective experience in those who perceive them.

## Significance Statement

The perception of emotion in others often results in related sensory experiences in oneself, which is thought to facilitate the social spread of emotions. Using functional neuroimaging, we have discovered a neural mechanism capable of explaining how percepts of emotion bias subjective experience. We show that activity in right somatosensory cortex can be used to classify emotions conveyed in facial and vocal expressions. Importantly, the capacity of this region to predict perceived emotions in others correlates with reports of subjective experience generated by the expressions in oneself. The results reveal a novel, specialized role for the somatosensory cortex in linking emotional perception with subjective sensory experience.

## Introduction

During social interactions, humans recognize emotions from facial and vocal cues with seemingly little effort. Often, the perception of emotions in others leads to the spread of emotional behaviors, such as crying and laughter (Provine, 1992). Simulationist models of emotion recognition (Adolphs, 2002; Goldman and Sripada, 2005; Niedenthal, 2007) propose that these phenomena result from neural processing in somatosensory cortex (Keysers et al., 2010). Although this region is primarily involved in tactile sensation, it has been argued that somatosensory representations also facilitate emotion recognition by linking nontactile perceptual cues to bodily states associated with each emotional category (Damasio, 1996).

Damage to or inactivation of right somatosensory cortex disrupts the recognition of emotion from facial (Adolphs et al., 2000; Pitcher et al., 2008) and vocal (Adolphs et al., 2002; Banissy et al., 2010) expressions. However, it is not known whether this behavioral impairment is due to an experiential mirroring mechanism, as suggested by embodied cognition perspectives. Although emotional expressions can be decoded from patterns of activation within unimodal (Ethofer et al., 2009; Harry et al., 2013) or multimodal association cortices (Peelen et al., 2010; Wegrzyn et al., 2015), it is unknown whether neural activity within somatosensory cortex codes categorical information from perceived emotions in the nontactile domain, and whether such activity is related to subjective sensory experience in terms of its separability and topographic organization.

To bridge this conceptual gap, we conducted a functional magnetic resonance imaging (fMRI) experiment in which participants were presented with facial and vocal expressions of discrete emotions and made on-line ratings of their own subjective experience in response to these percepts. This procedure offers insight into how emotional expression perception alters sensory experience as a component of affect, although emotional events occurring in everyday life or those elicited by laboratory mood inductions generally yield more rigorous, full-blown emotional experiences. Given that expressions of emotion lead to the convergence of facial configuration and shared mood (Hess and Blairy, 2001), we expected behavioral self-report to mirror the emotional content of stimuli. Further, if somatosensory representations reflect how one would feel when making an emotional expression, then it should be possible to decode emotion-specific patterns of neural activation within right somatosensory cortex, and the spatial configuration of these patterns should be consistent with known somatotopy.

## Materials and Methods

### Participants

Twenty-one healthy, right-handed individuals (*M*_age_ = 26 years, age range = 19–39 years, 11 males) completed the study. One additional participant was run in the experiment, but was excluded from analysis due to excessive head-motion during scanning (total displacement exceeding 1 cm). All participants provided written informed consent to participate in accordance with the Duke University Institutional Review Board and received $20/h as monetary compensation.

### Experimental paradigm

During scanning, participants were presented with facial and vocal expressions of emotion, followed by self-report. To isolate neural responses to the expressions, the period between stimulus presentation and motor response was jittered following a Poisson distribution (λ = 4 s).

The stimuli used included standardized images of faces (Langner et al., 2010) and audio recordings of pseudo-utterances (Pell et al., 2009), which convey emotions of happiness, surprise, fear, anger, and sadness, in addition to neutral control expressions. Twelve expressions were presented in each modality for each emotion, resulting in a total of 144 unique stimuli. Participants viewed stimuli in one of four pseudorandom counterbalanced orderings, which alternated between blocks of facial or vocal expressions. Each block consisted of one male and one female presentation of each emotion, totaling 12 trials. Facial stimuli were presented for 1.5 s, whereas auditory stimuli lasted 1.65 ± 0.32 s (mean ± SD). Each experimental session comprised three runs of data acquisition, including four blocks and lasting on average approximately 10.26 min.

During the self-report phase, the Geneva Emotion Wheel (Scherer, 2005) was presented on the screen for 6 s. This self-report assay contains 16 emotion words organized radially about the center of the screen, in a fixed position. Four circles emanate from the center of the screen to each word (similar to a spoke of a wheel), which can be used to indicate the intensity of each emotion. Participants were instructed to use a joystick to move the cursor from the center of the screen to the location on the screen that best indicated how they currently feel. Participants were told to move the cursor to the center of the screen if they did not feel any of the emotions listed. Prior to scanning, participants completed a set of practice trials wherein they moved the cursor to each emotion term, insuring functionality of the joystick and comprehension of the task.

Presentation of stimuli and acquisition of behavioral responses were controlled using Cogent 2000 software (Wellcome Department of Imaging Neuroscience, http://www.vislab.ucl.ac.uk/cogent.php). Participants viewed stimuli on mirrors aligned with a LCD screen upon which images were projected from a stimulus control computer. Audio stimulation was presented using MR-compatible headphones (Resonance Technology).

### Image acquisition

Scanning was performed on a 3 Tesla General Electric MR 750 system with 50-mT/m gradients and an eight-channel head coil for parallel imaging (General Electric). Structural images were acquired using a 3D fast SPGR BRAVO pulse sequence: repetition time (TR) = 7.58 ms; echo time (TE) = 2.936 ms; image matrix = 256^2^; α = 12°; voxel size = 1 × 1 × 1 mm; 206 contiguous slices) for coregistration with the functional data. Structural images were aligned in the near-axial plane defined by the anterior and posterior commissures. Whole-brain functional images were acquired using a spiral-in pulse sequence with sensitivity encoding along the axial plane (TR = 2000 ms; TE = 30 ms; image matrix = 64 × 128; α = 70°; voxel size = 3.8 × 3.8 × 3.8 mm; 34 contiguous slices). The first five images of each run were excluded from analyses to ensure the magnet had reached steady state.

### Preprocessing and estimating neural activation

Processing of MR data was performed using Statistical Parametric Mapping software (SPM8; Wellcome Department of Imaging Neuroscience). Functional images were slice-time-corrected, spatially realigned to correct for motion artifacts (Friston et al., 1995), coregistered to anatomical scans (Collignon et al., 1995), and normalized to Montreal Neurologic Institute (MNI) space using high-dimensional warping implemented in the VBM8 toolbox (http://dbm.neuro.uni-jena.de/vbm.html). Functional data were not spatially smoothed.

Whole-brain patterns of neural activation were estimated using the general linear model approach implemented in SPM8. For each subject, blood oxygen level-dependent (BOLD) responses were modeled by convolving box-car functions with a canonical hemodynamic response function separately for each trial. One additional regressor modelling the self-report phase was included in each run. To model nuisance effects, six motion parameters (roll, pitch, yaw, in addition to translation in *x*, *y*, and *z* dimensions) and session constants, were incorporated into the model.

### Regions-of-interest

Anatomical masks were created for brain regions implicated in a neural network model of emotion recognition (Adolphs et al., 2000, 2002) using the Automated Anatomical Labeling atlas (Tzourio-Mazoyer et al., 2002). In particular, masks were created for right postcentral gyrus (somatosensory cortex) and bilaterally for posterior superior temporal sulcus (defined as voxels within superior temporal gyrus posterior to *y* = −32 mm in MNI space), medial orbitofrontal cortex, inferior frontal operculum, fusiform gyrus, amygdala, and insula. For motor control analyses and lateralization tests, masks for left and right precentral and left postcentral gyrus were additionally used.

### Classification of self-report

Cursor locations (2-dimensional data centered on the center pixel on the presentation computer) were used to predict the emotional content of stimuli. This data-driven approach was employed because it does not assume fixed stimulus–response mappings for all subjects and provides classification weights to assess the consistency of mappings. Because the self-report format was circular in nature, classification was performed using support vector machines (SVMs) with a radial basis function as implemented in LIBSVM (Chang and Lin, 2011). As cursor locations were assigned one of six labels, the default “one-against-one” multiclass algorithm was used (Hsu and Lin, 2002). Nested fivefold cross validation was performed separately for each subject. The inner folds were implemented for selection of parameters *C* and γ, and the outer folds were used to provide cross-validated measures of accuracy. Because distributions of classification accuracy typically violate the assumptions of parametric tests, one-tailed Wilcoxon sign-rank tests were performed for group inference. To examine which cursor locations led to the prediction of each expression, one-sample *t* tests were performed across subjects on SVM decision values for all coordinates in the grid.

### Multivoxel pattern classification

Decoding of neural activity was performed via PLS-DA (Wold et al., 2001) using the NIPALS algorithm (Martens and Naes, 1989) as implemented in the libPLS toolbox (http://www.libpls.net). This method was selected because it effectively reduces the dimensionality of data, decreasing chances of overfitting.

Classification was performed using trials including both facial and vocal expressions, in order to identify emotion-specific patterns of neural activity that generalize across modalities. This approach is well suited to identifying embodied representations of emotion because it discourages learning low-level features of expressions (eg, fundamental frequency of vocalizations or visual contrast in facial expressions). Thus, the learning scheme emphasizes information that is independent of stimulus modality and should be more sensitive in detecting somatic states associated with facial and vocal cues. To ensure that the classifier was not biased toward stimuli of one modality, accuracies were compared for facial versus vocal expressions of emotion.

Classification of multiple categories was performed using a winner-takes-all approach, wherein one class is classified against all others. Because this approach creates an uneven proportion of classes (1:5), a weighted approach was taken for discriminant analysis to minimize bias due to class imbalance. Input data (144 trials) were mean-centered before conducting the analysis. The number of latent variables was fixed at 1, to reduce the complexity of the model, simplify interpretation of model coefficients, and maximize the amount of data available for training and testing. Classification was performed separately for each subject, using cross validation (interleaving trials between the two folds). Randomization of single trial estimates in the wavelet domain (Bullmore et al., 2001) was conducted to confirm that this cross-validation did not introduce a positive bias. This test confirmed that autocorrelation in the signals was not predictive, as classification of these scrambled data yielded an accuracy of 16.7 ± 3% (mean ± SD within regions, chance = 16.67%). Group inference on accuracy was performed using one-tailed Wilcoxon sign-rank tests (with chance rates of 1/6), with FDR correction (Benjamini and Hochberg, 1995) for multiple comparisons when appropriate.

To assess the relationship between experiential ratings and the information content of neural activation patterns, the accuracy of classifying self-report was correlated with the accuracy of classifying fMRI data across subjects for each region of interest, using Pearson’s coefficient. Inference was performed for each region using the Student’s *t* distribution (two-tailed), with FDR correction for multiple comparisons. To identify which region best characterized individual differences in self-report ratings, linear regression models predicting the accuracy of self-report from accuracy of neural classification were estimated, and model log-likelihoods were used to compute Bayesian Information Criterion (BIC; Wagenmakers and Farrell, 2004) values (using the fitglm and aicbic functions in MATLAB). BIC values were converted to weights (wBIC), which were compared to determine evidence ratios for different regions.

Comparisons of PLS regression coefficients within the postcentral gyrus were assessed using a one-way ANOVA. These coefficients characterize a linear mapping between BOLD activation and the likelihood membership for each emotion category. Contrasts were made between emotions associated with movement of facial muscles in the lower (happiness, surprise) versus upper (fear, anger) portions of the face (Bassili, 1979; Smith et al., 2005) at the group level. Fearful expressions are considered to involve primarily the upper portion of the face based because a jaw drop (action unit 26) is not always included in its prototypical expression (Langner et al., 2010). Additionally, there are some common movements among expressions, such as lip parting and brow raising, potentially leading this contrast to underestimate differences across categories. AR(1) correction was applied to adjust for departures from sphericity (independence and homogeneity of variance).

Statistical maps were thresholded using a voxelwise threshold of *p* < 0.05 and extent of 21 voxels, which we determined to control the false-positive rate at α < 0.05 using Monte Carlo simulations on the present data (Forman et al., 1995). Because this thresholding approach has recently been shown to be susceptible to high type-I error rates (Eklund et al., 2015), we double-checked these results against permutation based methods (Winkler et al., 2014) and found similar results. To test the extent to which clusters observed in the group model were distinct, we performed randomization tests (randomly flipping the weights of the contrasts for upper vs lower expressions) over 10,000 iterations. On each randomization, we identified the number of significant clusters for the contrast of expressions involving lower versus upper portions of the face. Renderings of classification weights for lower versus upper face expressions were mapped to flattened and inflated models of the cortical surface (Van Essen, 2005) in the right hemisphere using Caret (Van Essen et al., 2001).

## Results

**Figure 1.**
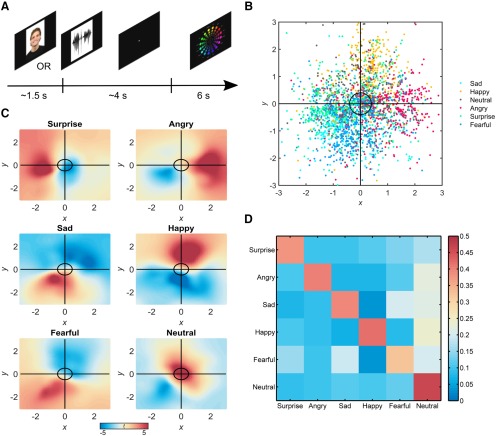
Experimental paradigm and behavioral results. ***A***, Graphical depiction of a single trial in which participants are first presented a facial or vocal expression of emotion, followed by a fixation cross, and a response screen, which subjects used to indicate their own emotional experience in response to the stimuli by moving a cursor. ***B***, Scatterplot of behavioral responses for all participants, with each point corresponding to a single trial. Axes reflect cursor positions along horizontal and vertical dimensions of the screen, standardized within subjects. ***C***, Parametric maps (one sample *t* test, *n* = 21) of support vector machine decision values for each emotion category, showing which coordinates lead to the prediction of each emotion. Cursors located in blue regions are evidence against the labeled category, whereas red regions indicate positively predictive regions. ***D***, Confusion matrix for classification of self-report. Color bar indicates proportion of trials (chance = 16.67%) from each emotion category (rows) assigned each label during classification (columns).

We first tested whether participants’ reported sensory experience was consistent with those conveyed by facial and vocal expressions by constructing classification models to predict the emotional content of stimuli using cursor locations on every trial. Consistent with our hypothesis of behavioral mirroring, cursor positions spanning both facial and vocal trials demonstrated significant discrimination, with an accuracy of 40.1 ± 3.79% (mean ± SEM), compared to chance levels of 16.67% (Wilcoxon sign-rank test, *z* = 3.84, *p* = 1.22e-04)^a^ (Note: superscript letters refer to statistical tests indexed in Table 1.). Examination of decision values from the classifiers revealed that emotions were best predicted by ratings within focal regions of the self-report inventory (Fig. 1*C*), indicating that participants experienced relatively discrete sensations in response to the facial and vocal stimuli. Together, these findings provide clear evidence that participants’ self-reported experiences were congruent with those perceived from the facial and vocal cues.

**Figure 2.**
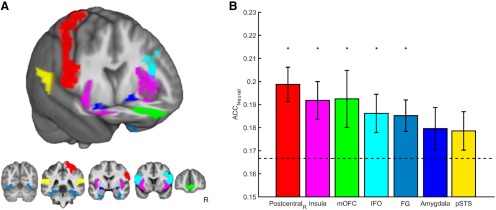
Multivoxel pattern classification of BOLD response to facial and vocal expressions of emotion. ***A***, ROIs rendered on the group mean anatomical image (*n* = 21). ***B***, Patterns of response within right postcentral gyrus (*z* = 3.21, *p*_adj_ = 0.0047)^b^, insula (*z* = 2.66, *p*_adj_ = 0.0136)^c^, mOFC (*z* = 1.92, *p*_adj_ = 0.0384)^d^, IFO (*z* = 1.93, *p*_adj_ = 0.0384)^e^, and FG (*z* = 2.43, *p*_adj_ = 0.0175)^f^ were classified at levels greater than chance (Wilcoxon sign-rank test). Dashed line reflects chance accuracy (16.67%). Error bars reflect SEM. ACC = accuracy.

To examine whether regional patterns of fMRI response discriminated among perceived emotions, we conducted multivoxel pattern classification on data from brain regions implicated in a neural network hypothesized to be critical for the recognition of emotion (Adolphs, 2002): postcentral gyrus in the right hemisphere (corresponding to primary somatosensory cortex), posterior superior temporal sulcus (pSTS), medial orbitofrontal cortex (mOFC), inferior frontal operculum (IFO), fusiform gyrus (FG), amygdala, and insula. Among these regions, decoding of emotional categories from perceptual cues was successful from patterns of activation in postcentral gyrus, mOFC, IFO, FG, and insula at accuracy levels significantly above chance (all *p*_adj_ < 0.05; Fig. 2)^b–h^, although they were near the chance distribution’s margin of error. Differences in classification accuracy between facial and vocal expressions did not reach statistical significance in any region (all *p*_adj_ > 0.10)^i–o^, indicating that learning was not generally biased toward either modality.

Although the right postcentral gyrus exhibited the highest accuracy level at 19.9 ± 0.75% (mean ± SEM), follow-up comparisons did not reveal significant differences between right somatosensory cortex and any other region-of-interest (ROI; all *p*_adj_ > 0.09)^p–u^. Given evidence specifically implicating the right somatosensory cortices in emotion recognition (Adolphs et al., 2000, 2002), we compared classification accuracy in left and right postcentral gyrus. This analysis revealed a moderate effect for higher accuracy in the right hemisphere, although it was only marginally significant (two-tailed Wilcoxon sign-rank test; *z* = 1.95, *p* = 0.0507)^v^.

Having established that patterns of fMRI activity within right somatosensory cortex predict the emotional content of facial and vocal expressions in a manner consistent with self-reported experience, we next tested whether classification weights within this region followed somatotopic organization consistent with those of perceived emotions. Although the spatial resolution of fMRI is too coarse to directly sample neural activity sensitive to individual facial muscles, and there may be some common facial movements involved in different emotions, we postulated that the overrepresentation of the lip, cheek, and mouth regions in somatosensory cortex could be used to compare emotional expressions that differentially engage lower versus upper regions of the face.

**Figure 3.**
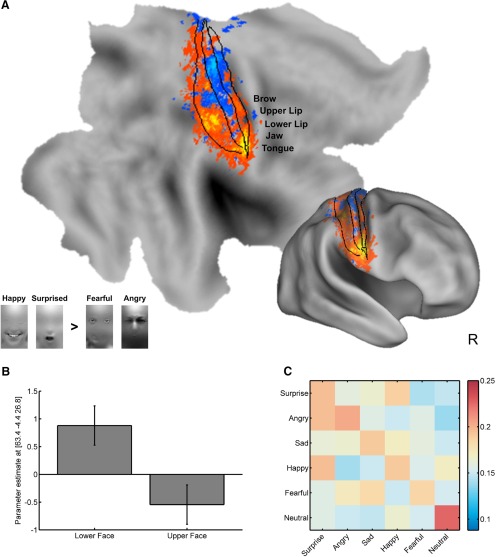
Emotion-predictive patterns are consistent with known somatotopy. ***A***, Contrasts of classification weights reveal the perception of expressions associated with lower portions of the face was predicted by greater activation in inferior regions of the postcentral gyrus. Solid lines demarcate borders of BAs 3, 1, and 2. Text overlays indicate hypothesized somatotopy from upper to lower regions of the face. Inset of facial images convey portions of the face that are diagnostic of each expression (adapted with permission from Smith et al. 2005). ***B***, Contrasts of parameter estimates show that activation near the lateral sulcus selectively predicts expressions of happiness and surprise (lower face emotions) relative to fear and anger (upper face emotions). Error bars reflect 95% confidence intervals based on within-subject error (Cousineau, 2005). ***C***, Mean confusion matrix depicts classifications based on somatosensory data (columns) against true class labels (rows). Higher values along the main diagonal illustrate above-chance performance (chance = 16.67%). Confusions between happiness and surprise are consistent with somatotopic patterning driven by activity associated with lower portions of the face and mouth. Color bar indicates proportion of predictions (rows sum to one).

Because prior research has shown that happiness and surprise contain more distinctive information in lower regions of the face, we speculated that expressions of happiness and surprise would have larger classification weights than those of fear and anger, which contain more distinguishing information in upper portions of the face (Bassili, 1979; Smith et al., 2005). This exploratory analysis revealed two clusters in lateral postcentral gyrus (Fig. 3*A*); one cluster spanned Brodmann areas (BAs) 3, 1, and 2 adjacent to parietal operculum (MNI center of mass = 57, −6, 28; peak *t*_(20)_ = 3.15)^w^ whereas the other was restricted to BA 2 (MNI center of mass = 40, −30, 46; peak *t*_(20)_ = 3.65)^x^. The localization of these peaks is consistent with studies localizing oral and facial (Miyamoto et al., 2006; Eickhoff et al., 2008) representations in somatosensory cortex. We performed randomization tests to assess the probability of observing two separate clusters (see Materials and Methods). Over 10,000 iterations, only 50 times did a single cluster exceed the corrected threshold of *p* < 0.05, *k* > 20 voxels (*p* = 0.005); two clusters were never observed (*p* = 0.00001). These results demonstrate that expressions of happiness and surprise, compared to fear and anger, were predicted by activity in two distinct clusters in the postcentral gyrus.

**Figure 4.**
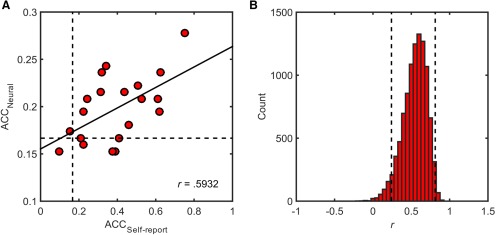
The information content of response patterns within right postcentral gyrus increases with the separability of self-report. ***A***, Scatterplot depicts cross-validated estimates of accuracy across all emotion categories for classification of self-report and neural data, with each point corresponding to a single subject (*n* = 21). Solid black line indicates the best least-squares fit to the data. Dashed lines reflect chance accuracy (16.67%). ***B***, Histogram of bootstrap distribution of Pearson’s correlation coefficient, with dashed lines indicating 95% confidence interval computed using the bias corrected and accelerated percentile method. ACC = accuracy.

Given that pattern classification is opportunistic in discriminating among brain states and may have been driven by factors other than experienced emotion *per se* (eg, low-level stimulus properties or physiological arousal), we next tested whether individual differences in the accuracy of neural classification correlated with those of self-report. We found that the degree to which individuals reported distinct sensory experiences was uniquely associated with the information content of patterns spanning the full extent of postcentral gyrus (*r* = 0.5932, *p*_adj_ = 0.041^y^; *p*_adj_ > 0.2 for all other regions; Fig. 4; Table 2). Bayesian comparisons of these linear associations revealed that somatosensory cortex was >12 times more likely to predict individual differences in sensory experience than the next most likely brain region, the pSTS. Differences in BIC values strongly favored the somatosensory model against all other models (ΔBIC > 6; Kass and Raftery, 1995), with the exception the pSTS model, which still showed positive support for the somatosensory model (ΔBIC = 5.02). Such a strong correspondence establishes a direct link between the information content of somatosensory activity and self-reported experience during the perception of facial and vocal expressions of emotion.

**Table 2.** Correlations between neural and self-report classification accuracy

**ROI**	**Pearson’s *r***	***p* Adjusted**	***p* Uncorrected**	**BIC**	**ΔBIC**	**wBIC**
Right postcentral gyrus	0.593	0.041	0.005	−21.020	0.000	0.833
pSTS	0.420	0.260	0.058	−15.996	5.024	0.068
mOFC	0.219	0.510	0.340	−12.944	8.076	0.015
IFO	0.257	0.510	0.261	−13.347	7.673	0.018
FG	0.085	0.802	0.713	−12.066	8.954	0.009
Amygdala	0.128	0.747	0.581	−12.257	8.763	0.010
Insula	0.051	0.826	0.826	−11.967	9.053	0.009
Left precentral gyrus	0.287	0.510	0.208	−13.712	7.308	0.022
Right precentral gyrus	0.233	0.510	0.309	−13.085	7.936	0.016

**Table 1.** Statistical table

	**Comparison**	**Data structure**	**Type of test**	**Observed power**
a	Classification accuracy: self-report; average vs chance	Binomial	Wilcoxon sign-rank test (against constant)	0.9620045
b	Classification accuracy: right postcentral gyrus; average vs chance	Binomial	Wilcoxon sign-rank test (against constant)	0.9565211
c	Classification accuracy: insula; average vs chance	Binomial	Wilcoxon sign-rank test (against constant)	0.8235641
d	Classification accuracy: medial OFC; average vs chance	Binomial	Wilcoxon sign-rank test (against constant)	0.5337574
e	Classification accuracy: inferior frontal operculum; average vs chance	Binomial	Wilcoxon sign-rank test (against constant)	0.5378919
f	Classification accuracy: fusiform gyrus; average vs chance	Binomial	Wilcoxon sign-rank test (against constant)	0.7418888
g	Classification accuracy (objective labels): amygdala; average vs chance	Binomial	Wilcoxon sign-rank test (against constant)	0.3522203*
h	Classification accuracy (objective labels): posterior STS; average vs chance	Binomial	Wilcoxon sign-rank test (against constant)	0.4092183*
i	Classification accuracy (objective labels): postcentral gyrus; facial vs vocal	Binomial	Wilcoxon sign-rank test (paired)	0.0789112*
j	Classification accuracy (objective labels): insula; facial vs vocal	Binomial	Wilcoxon sign-rank test (paired)	0.6379089*
k	Classification accuracy (objective labels): medial OFC; facial vs vocal	Binomial	Wilcoxon sign-rank test (paired)	0.130852*
l	Classification accuracy (objective labels): inferior frontal operculum; facial vs vocal	Binomial	Wilcoxon sign-rank test (paired)	0.7368259*
m	Classification accuracy (objective labels): fusiform gyrus; facial vs vocal	Binomial	Wilcoxon sign-rank test (paired)	0.0578589*
n	Classification accuracy (objective labels): amygdala; facial vs vocal	Binomial	Wilcoxon sign-rank test (paired)	0.0532535*
o	Classification accuracy (objective labels): posterior STS; facial vs vocal	Binomial	Wilcoxon sign-rank test (paired)	0.0535096*
p	Classification accuracy (objective labels): right postcentral gyrus vs insula	Binomial	Wilcoxon sign-rank test (paired)	0.1202871*
q	Classification accuracy (objective labels): right postcentral gyrus vs medial OFC	Binomial	Wilcoxon sign-rank test (paired)	0.1961514*
r	Classification accuracy (objective labels): right postcentral gyrus vs IFO	Binomial	Wilcoxon sign-rank test (paired)	0.4217805*
s	Classification accuracy (objective labels): right postcentral gyrus vs fusiform gyrus	Binomial	Wilcoxon sign-rank test (paired)	0.5747844*
t	Classification accuracy (objective labels): right postcentral gyrus vs amygdala	Binomial	Wilcoxon sign-rank test (paired)	0.6903970*
u	Classification accuracy (objective labels): right postcentral gyrus vs posterior STS	Binomial	Wilcoxon sign-rank test (paired)	0.8736939*
v	Classification accuracy (objective labels): right vs left postcentral gyrus	Binomial	Wilcoxon sign-rank test (paired)	0.6693379
w	PLS regression coefficients (objective labels): upper vs lower face emotions	Normal	One-sample *t* test	0.9999976
x	PLS regression coefficients (objective labels): upper vs lower face emotions	Normal	One-sample *t* test	1.0000000
y	Classification accuracy (objective labels): self-report against right postcentral gyrus	Binomial	Correlation (Pearson)	0.9092103
z	Classification accuracy (subjective labels): right postcentral gyrus	Binomial	Wilcoxon sign-rank test (against constant)	0.9974600
aa	Classification accuracy: objective vs subjective labels, right postcentral gyrus	Binomial	Wilcoxon sign-rank test (paired)	0.0500000
bb	PLS regression coefficients: objective against subjective models	Binomial	One-sample *t* test (Fisher transformed correlation)	0.9999025
cc	Classification accuracy: left precentral gyrus; average vs chance	Binomial	Wilcoxon sign-rank test (against constant)	0.0684199
dd	Classification accuracy: self-report against left precentral gyrus	Binomial	Correlation (Pearson)	0.3588166
ee	Classification accuracy: postcentral gyrus; average vs chance	Binomial	Wilcoxon sign-rank test (against constant)	0.9830124
ff	Classification accuracy: self-report against right precentral gyrus	Binomial	Correlation (Pearson)	0.2688632

Data are assumed to come from the stated distributions. For sign-rank tests, effect sizes are computed as *r* = z/√n, from which achieved power is calculated. *Effects that were not significant (correcting for multiple comparisons) and were not included in the main text of the paper.

To dissociate subjective representations elicited by the stimuli from simple encoding of emotion categories, we constructed classification models to predict emotion categories defined on the basis of self-report. Classifying somatosensory activity using self-report ratings in lieu of stimulus categories produced similar results: mean accuracy was 19.74 ± 0.82% (SEM; *z* = 3.25, *p* < 0.0011)^z^, which did not significantly differ from classification accuracy based on stimulus categories, (*p* = 1, signed-rank test)^aa^. To assess the extent to which classification utilized independent information, the correlation between classification weights from objective and subjective models was computed within subjects and averaged across all six emotions. This analysis revealed a moderate correlation [*r* = 0.3115 ± 0.052 (SEM), *p* = 0.000019]^bb^, suggesting that subjective experience and objective stimulus category are reflected in at least some shared variance in somatosensory response patterns, although the amount of reliable, unique variance attributed to each remains to be determined.

Given the strong interconnections between corresponding sensorimotor areas of precentral and postcentral gyrus, and evidence that neurons in both cortical areas respond during motor or sensory behavior (Mouret and Hasbroucq, 2000), we conducted control analyses in motor cortices using left and right precentral gyrus ROIs to rule out an alternative interpretation that the present results are related to motor preparation (as the emotion labels were presented in fixed locations) or motor feedback. Decoding performance in left motor cortex was not significantly different than chance levels with 18.0 ± 1.0% accuracy (mean ± SEM; chance = 16.67%, *z* = 1.43, *p* = 0.0751)^cc^, making it unlikely that motor activity in preparation of moving the joystick drove results. Additionally, activity in this region was not associated with self-report (*r* = 0.287, *p* = 0.208)^dd^. Although voxel patterns in right motor cortex were found to predict the emotional content of stimuli with 19.0 ± 0.59% accuracy (mean ± SEM; *z* = 3.21, *p* = 0.0013)^ee^, they did not correlate with experiential ratings (*r* = 0.233, *p* = 0.3094)^ff^. Bayesian analysis revealed the association between the accuracy of self-report and neural activation within somatosensory cortex was much more likely than for classification of left or right precentral gyrus activity (evidence ratios of 38.6 and 52.9, respectively).

## Discussion

Our results demonstrate that patterned activation within somatosensory cortex contains information sufficient for the decoding of perceived emotional categories. Such refined discrimination of nontactile stimulation within somatosensory cortex runs contrary to the classic view that the region is a unimodal sensory area and suggests that visual and auditory signals modulate neural activity at early stages of cortical processing in this region (Ghazanfar and Schroeder, 2006). Additionally, the localization of effects in the right hemisphere is in general agreement with models of asymmetric emotional processing based on lesion studies (DeKosky et al., 1980; Ross, 1981; Blonder et al., 1991; Borod, 1992), although it is important to note that fMRI has lower sensitivity and specificity when testing for lateralization (Ross and Monnot, 2008). Together, our findings expand the functional role of the somatosensory cortex and provide novel evidence that emotions are reflected partly in the brain’s representation of the body (Damasio, 1996; Adolphs, 2002; Niedenthal, 2007).

In an exploratory analysis, we found that emotion-predictive patterns within postcentral gyrus exhibited somatotopic organization, suggesting that information related to body states contributed to the decoding of emotional expressions. This result is concordant with evidence that emotions are associated with categorically distinct bodily sensations (Nummenmaa et al., 2014). Further, the small number of classification errors among negative emotions (Fig. 3*C*) demonstrates that factors beyond valence (Russell et al., 2003) organize somatosensory activity, although this conclusion warrants further investigation as happiness was the only positive emotion sampled. Until a broader array of emotions are tested, it remains possible that some combination of valence, arousal, or approach-withdrawal motivation better explain the observed somatotopy. The confusion matrix additionally revealed relatively few errors for neutral expressions, a finding consistent with classification of self-reported bodily sensations (Nummenmaa et al., 2014), distributed brain responses to emotional experiences (Kragel and LaBar, 2015), and dynamic facial expressions of emotion (Said et al., 2010).

Topographically organized somatosensory activation has been documented during the observation of touch (Blakemore et al., 2005; Ebisch et al., 2008; Schaefer et al., 2009), during the observation of actions (Gazzola and Keysers, 2009), and during the perception of sound (Gazzola et al., 2006). Given that BAs 3 and 1 are more closely tied to tactile stimulation, whereas BA 2 has generally been implicated in processing proprioceptive information (Keysers et al., 2010), our localization of emotion-predictive patterns in all three areas suggests that a combination of tactile and proprioceptive information is simulated during the perception of emotional expressions. Although our localization of upper versus lower face representation is broadly in agreement with the known somatosensory homunculus, attempts at validation against independent studies were challenging, as there is no available human somatotopic atlas, and the small number of studies comparing different regions of the face use different stimulation and normalization procedures and have mixed results (for review, see Haggard and de Boer, 2014). Due to the many challenges involved in precise somatotopic mapping of the face, including head-motion constraints, the need for a specialized head coil, mechanoreceptor receptive field sizes, and variability in individual anatomy, the present results should be considered preliminary as more precise mapping to specific facial regions is left as a future direction.

Beyond predicting the emotional content of stimuli, we found that somatic representations of perceived emotions uniquely correlated with the extent of experiential mirroring across individuals. The fact that this correlation was selective to somatosensory cortex suggests that factors influencing global levels of neural activity, such as arousal or attentiveness, were not likely the source of individual differences because they would lead to enhanced discriminability in other brain regions. Our observation of experiential mirroring is consonant with behavioral studies showing the perception of emotional expressions leads to facial mimicry and congruent self-reports (Hess and Blairy, 2001) and further supports accounts that posit emotion-related knowledge is embodied in somatosensory cortices (Goldman and Sripada, 2005; Niedenthal, 2007). Activation of emotion categories in somatosensory cortex may directly or may indirectly contribute to conscious experience through local processing or connections with distributed neural networks.

Given that emotions serve action preparation functions and involve motor feedback, it is important to consider the potential role of these functions as an explanation for the somatosensory findings. The observation that emotion-predictive patterning in primary motor cortex was not associated with behavioral self-report suggests that facial mimicry or other forms of motoric engagement, while potentially contributing to emotion recognition, was not likely responsible for the convergence of perception and subjective experience in the right postcentral gyrus. However, future work more precisely monitoring facial muscle activity will be necessary to definitively resolve this issue. Although null results should be interpreted with caution, this finding is in accordance with other studies that failed to identify a correspondence between facial mimicry and emotional feelings (Blairy et al., 1999; Hess and Blairy, 2001). By linking experiential ratings to distinct patterns of somatic activity, we provide a mechanistic interpretation for studies showing that primary somatosensory cortex plays an essential role in emotion recognition (Adolphs et al., 2000, 2002; Pitcher et al., 2008; Banissy et al., 2010) that is consistent with the somatic marker hypothesis (Damasio, 1996), wherein representations of body states associated with distinct emotions contribute to cognitive processing.

In addition to primary somatosensory cortex, we found that patterns of BOLD response within a number of regions implicated in emotion recognition predicted the emotional content of stimuli, but were not associated with individual differences in sensory experience. These regions are thought to process distinct kinds information associated with emotional expressions (Adolphs, 2002). The orbitofrontal cortex, for example, is widely implicated in the representation of subjective value (Clithero and Rangel, 2014), affective valence (Chikazoe et al., 2014), and responds to the attractiveness of faces; an effect which is modulated by the presence of a happy facial expressions (O'Doherty et al., 2003). The insula is broadly involved in interoceptive processing (Craig, 2002), responds to diverse affective cues (Sander and Scheich, 2001; Aubé et al., 2015), in particular to facial expressions of disgust (Phillips et al., 1997; Sprengelmeyer et al., 1998; Wicker et al., 2003; but see Phillips et al., 1998; Schienle et al., 2002). Although the insula is associated with numerous functions (Chang et al., 2013), activity in this region could ostensibly reflect interoceptive states associated with distinct emotions. Although the role of the fusiform gyrus in processing basic visual features of faces is relatively well characterized (Haxby et al., 2000), activation of this region has additionally been observed during the perception of emotional vocalizations (Rämä et al., 2001; Johnstone et al., 2006) and during semantic processing of auditory content (Chee et al., 1999). Thus, although our findings highlight the role of somatosensory cortex in subjective experience, we stress that other factors, such as subjective value, interoceptive processes, conceptual knowledge, and sensory and motor modulation, likely contribute to the perception of emotions in social signals as well.

Our methodological approach serves as a template for subsequent work examining the role of somatic states in socio-emotional behavior. Independent characterization of somatotopy at the single-subject level (Huang and Sereno, 2007) using high-resolution protocols (Meier et al., 2008; Sanchez-Panchuelo et al., 2010; Stringer et al., 2011) may provide more detailed characterization of somatosensory states associated with specific emotions. Assaying somatic states during the disruption of facial muscle activity (eg, Hennenlotter et al., 2009) could establish whether peripheral feedback is essential in producing the observed effects, or whether centrally generated representations of body states are sufficient. The frequency and separability of somatic states could further be quantified during live social interactions (Redcay et al., 2010), to characterize their occurrence in more ecologically valid settings. Future studies in these areas are necessary to characterize the role embodied emotions play in social interactions.

## Synthesis

The decision was a result of the Reviewing Editor Daniela Schiller and the peer reviewers coming together and discussing their recommendations until a consensus was reached. A fact-based synthesis statement explaining their decision and outlining what is needed to prepare a revision is listed below. The following reviewers agreed to reveal their identity: Thomas FitzGerald, Philipp Homan

The authors have done a nice job in improving the paper.

However, there are two issues that require the authors' attention:

The first issue concerns the analysis designed to show there are different peaks for happiness/surprise and fear/anger. The authors seem to have used bootstrap statistics within these contrasts. However, the key null hypothesis is surely that happiness/surprise and fear/anger are interchangeable, and the appropriate test would be to randomly permute the assignment of labels between conditions, and test whether observed peaks were farther apart than predicted by chance (or that they lie further apart along some spatial gradient than would be predicted by chance). The authors could either perform an analysis of this type, or remove this analysis from the paper. Since this is only a supplementary finding, removing it would not affect the key conclusions of the paper.

Second, the argument that the somatosensory cortex is specific in showing the correlation between neural and self-report accuracy relies on a model selection using the weighted Bayesian Information Criterion (BIC). This procedure is not convincing. Comparing BIC values would make sense to test competing models against some observed data (y), very often involving different numbers of predictors. Here, however, the authors test "models" with just one predictor that is different in each model to support the claim that one predictor is better than the others in predicting y. If the authors choose this procedure, they should include not only weighted BIC values but also the original BIC values together with the Deltas, which would make the assessment of these values easier (see Table 1 in the Comment by Wagenmakers and Farrell).

However, a straight forward approach to compare two correlations would be to simply compare the Pearson r values by using Fisher's r-to-z transformation and to test whether the z-value is greater than |1.96| which would mean significance in a 2-tailed test performed. It should be noted, however, that the correlation coefficients would have been obtained from the same sample, with both correlations sharing one variable in common (the accuracy in separating the emotions behaviorally), and thus a procedure should be used as suggested by J. Steiger (1980), "Tests for comparing elements of a correlation matrix", Psychological Bulletin, 87, 245-251.

It is unclear whether the authors will be able to show that the correlation is specific for the somatosensory cortex. It might be unlikely that the correlations of somatosensory cortex (r=.593) and pSTS (r=.42) will be significantly different; in fact, it is unclear whether there will be a significant difference between the correlation of motor cortex (r=.233) and somatosensory cortex (r=.593). Anyway, if the correlation of the somatosensory cortex is unspecific some passages in the manuscript might need additional editing to take that into account.
